# Mexican-origin women’s individual and collective strategies to access and share health-promoting resources in the context of exclusionary immigration and immigrant policies

**DOI:** 10.1186/s12889-024-19204-3

**Published:** 2024-07-02

**Authors:** Alana M.W. LeBrón, Amy J. Schulz, Cindy Gamboa, Angela Reyes, Edna Viruell-Fuentes, Barbara A. Israel

**Affiliations:** 1 Department of Health, Society, and Behavior, University of California, Irvine 856 Health Sciences Drive, Suite 3555, Irvine, CA USA; 2 Department of Chicano/Latino Studies, University of California, Irvine, Irvine, CA USA; 3 Department of Health Behavior and Health Education, University of Michigan School of Public Health, Ann Arbor, MI USA; 4 Detroit Hispanic Development Corporation, Detroit, MI USA; 5 Department of Latina/o Studies, University of Illinois Urbana-Champaign (Deceased), Urbana- Champaign, IL USA

**Keywords:** Immigration enforcement, Immigrant policies, Racialization, Racism, Health, Latino, Latina, Latiné, Mexican, Immigrant

## Abstract

**Background:**

A growing literature has documented the social, economic, and health impacts of exclusionary immigration and immigrant policies in the early 21st century for Latiné communities in the US, pointing to immigration and immigrant policies as forms of structural racism that affect individual, family, and community health and well-being. Furthermore, the past decade has seen an increase in bi-partisan exclusionary immigration and immigrant policies. Immigration enforcement has been a major topic during the 2024 Presidential election cycle, portending an augmentation of exclusionary policies towards immigrants. Within this context, scholars have called for research that highlights the ways in which Latiné communities navigate exclusionary immigration and immigrant policies, and implications for health. This study examines ways in which Mexican-origin women in a midwestern northern border community navigate restrictive immigration and immigrant policies to access health-promoting resources and care for their well-being.

**Methods:**

We conducted a grounded theory analysis drawing on interviews with 48 Mexican-origin women in Detroit, Michigan, who identified as being in the first, 1.5, or second immigrant generation. Interviews were conducted in English or Spanish, depending on participants’ preferences, and were conducted at community-based organizations or other locations convenient to participants in 2013–2014.

**Results:**

Women reported encountering an interconnected web of institutional processes that used racializing markers to infer legal status and eligibility to access health-promoting resources. Our findings highlight women’s use of both individual and collective action to navigate exclusionary policies and processes, working to: (1) maintain access to health-promoting resources; (2) limit labeling and stigmatization; and (3) mitigate adverse impacts of immigrant policing on health and well-being. The strategies women engaged were shaped by both the immigration processes and structures they confronted, and the resources to which they had access to within their social network.

**Conclusions:**

Our findings suggest a complex interplay of immigration-related policies and processes, social networks, and health-relevant resources. They highlight the importance of inclusive policies to promote health for immigrant communities. These findings illuminate women’s agency in the context of structural violence facing immigrant women and are particularly salient in the face of anti-immigrant rhetoric and exclusionary immigration and immigrant policies.

## Health implications of immigration and immigrant policies

The Centers for Disease Control and Prevention [1] officially declared racism a serious public health problem. Racialization processes involve the creation and maintenance of dominant and subordinate racial groups by systems, institutions, and individual agents [2, 3]. These processes, such as differential value assigned to racial groups, often manifest through discourse, policies, and practices that ultimately shape racial inequities in rights, opportunities, resources, and treatment [2, 3]. Racialization processes operate and are experienced within particular temporal, social, political, and geographic contexts [4, 5]. United States (U.S.) immigration and immigrant policies constitute an important system that (re)produces inequities in the rights, opportunities, treatment, and health of racially minoritized immigrant groups. In the early twenty-first century, Mexican-origin communities have been particularly affected by exclusionary policies and practices towards immigrants, with nearly 70% of deportations from the U.S. occurring among Mexican-origin immigrants [6]. Further, restrictive state-level policies have flourished, with implications for social, economic, health care, and health outcomes for immigrant communities [7]. Furthermore, over the past decade, national, state and local actors have implemented increasingly restrictive immigration enforcement efforts and exclusionary immigrant policies [8–10]. Immigration enforcement has been a major topic during the 2024 Presidential election cycle, portending an augmentation of exclusionary policies towards immigrants [11, 12]. Together, these federal and state level policies and practices constitute a powerful form of structural racism [8, 13]. Understanding individual and collective experiences and responses to these processes, within particular contexts, is central to unpacking their implications for health equity. We examined the experiences of Mexican-origin women and the strategies they used to access health-promoting resources that are circumscribed in their northern border community of Detroit, Michigan. We discuss implications for health and health inequities, during a time of increasingly exclusionary immigration and immigrant policies and practices.

U.S. immigration policies affect opportunities to lawfully migrate to and live in the U.S. [14]. Historically and contemporarily, such policies have contributed to the development of the construct of “illegal” immigration statuses, the growth of border enforcement apparatuses, and quotas and other mechanisms that prioritize or limit migration to the U.S. according to country or region of origin [15]. In the twenty-first century, exclusionary immigration policies have taken the form of increased border enforcement systems and actions, detention of immigrants, and increasing collaborations between federal immigration enforcement agencies and local law enforcement in the U.S. interior [16–18]. The literature regarding the impacts of deportation, immigration raids, immigrant detention, and concerns about deportation details several pathways by which these policies and practices shape the health of Latiné communities (a gender-inclusive term referring to peoples of Latin American origin or descent; “Latines” when used in the plural). Pathways include, for example: family separation, which removes loved ones and income-earners from households and kin networks [19]; income loss and foreclosures [20]; and restricted access to healthy foods [19]. Additional pathways to poor health outcomes include chronic and acute stress related to immigration enforcement actions and deportation threat [19, 21, 22], and restricted access to and distrust in health care and public health resources [22–25]. A growing literature has linked exclusionary immigration and immigrant policies to adverse birth outcomes [26], cardiometabolic conditions [27], mental well-being [21, 28]– [30], and self-rated health [10]. Immigrant policies, or policies regulating the lives of immigrants based on legal status, impact health through their impacts across multiple sectors, including health care, government-issued ID, education, law enforcement, employment, and social welfare [8, 14, 19, 31]– [37].

State-level policies have been an important site of immigrant policy development in the twenty-first century, with important consequences for access to social and economic resources linked with health. For example, Young and colleagues [7] found that among Latines, U.S.-born and naturalized citizens experienced more favorable socioeconomic statuses in states with more inclusionary policies towards immigrants. This benefit did not extend to non-citizen Latines, suggesting that the space between inclusive policies, practices, and treatment towards non-citizens may be a persistent space of racial inequities. Almeida and colleagues (2016) found that state-level exclusionary immigrant policies were associated with higher levels of self-reported discrimination among Latines [38]. Also, following the passage of SB 1070, a multiple-measure exclusionary immigrant policy in Arizona, Toomey and colleagues [39] reported that Mexican-origin families used fewer preventive health care resources and public assistance. As another example, scholars have identified exclusionary state identification or driver’s license policies – in response to the federal REAL ID ACT of 2005 – as immigrant policies that constrain access to health-promoting resources increasingly linked with having current government-issued ID [40–43]. The REAL ID Act now has a national implementation deadline of 2025 [44]: Following that point, state IDs will need to meet more stringent criteria to be recognized for national identification purposes (e.g. airplane flights). As components of racialization processes, the immigration and immigrant policies described above not only contribute to constrained access to health-promoting resources, they also stigmatize Latiné and immigrant identities invoked in immigration and immigrant policy discourse and practice [43, 45]– [48]. Racially minoritized individuals and communities find ways to leverage their power and agency to respond to and navigate racialization processes [43, 49, 50]. Although substantial scholarship to date has documented effects of restricted access to health-promoting resources on the health of Latiné immigrant communities [8, 14, 25, 51, 52], variations in how Latines navigate this restricted access, experiences of discrimination, and strains on social networks within particular temporal political, social and geographic contexts are poorly understood. The aim of this study is to address this gap in the literature, and to examine implications for health.

## Immigration and immigrant policies, social networks, and health

The literature regarding social networks, social support, and the health of U.S. Latiné immigrant communities highlights the role of both transnational ties and relationships with U.S.-based networks in giving and receiving support: Scholars working within this area have suggested that leveraging and providing social support is a contextually-specific, negotiated process [53–57]. Given the gendered nature of social support and health-related support (both seeking and conferring), this literature largely focuses on the experiences of women [58]. Previous scholars have reported that for Mexican-origin women, social ties and social support were contingent upon immigrant generation and social context [54]. Viruell-Fuentes and Schulz (2009) reported that while some women in this study described drawing material, informational, and emotional support from close ties, others – particularly first-generation immigrant women – also described immigration-related isolation, tensions within kin networks, and concerns about depleting limited network resources [54]. Among women who had the means and resources to invest in building relationships with others outside of their immediate social networks, social ties established through those efforts enabled women to contribute to and access other social resources and enhanced their sense of belonging [54]. In contrast, second-generation women reported social ties that linked them to positive co-ethnic histories and kinship networks that were critical for affirming racial identities that were stigmatized in U.S. contexts [54]. The aim of this study is to illuminate how first- and second-generation Mexican-origin women leverage social support and their networks as they navigate restricted access to health-relevant resources.

### Immigration and immigrant policy context in Detroit, Michigan

The study described below focuses specifically on the experiences of Mexican-origin women and their social networks in Detroit, Michigan, as they navigate exclusionary federal- and state-level immigration and immigrant policy contexts unfolding in their border community, which has experienced a legacy of race-based residential segregation [59]. Border enforcement and interior immigration enforcement have increased in Michigan, a midwestern border state, in the twenty-first century [43]. Detroit is a border town, with an international bridge to Canada that cuts through Southwest Detroit, a neighborhood that is home to a longstanding Latiné, immigrant, and low-income community [60]. In 2008, in response to the REAL ID Act of 2005, the state began denying access to state-issued IDs or driver’s licenses to residents who could not prove their authorized U.S. presence [61]. Our past scholarship found that in the years following the implementation of this policy and practices to deny access to state-issued IDs in Michigan, many Mexican-origin immigrant women with whom we interviewed and their social networks in Detroit experienced a transformative loss of their Michigan driver’s licenses when clerks began scrutinizing their legal status as they applied for or renewed a driver’s license [43, 50]. Concurrent with restricted access to state-issued IDs in Detroit, government-issued IDs also became increasingly required to access health-promoting resources such as medications, housing, banking, and health care [32, 41, 62]. While our past scholarship largely focused on how these racialization processes unfolded and women’s strategies to navigate racialization processes [43, 50], the present study focuses explicitly on how Mexican-origin women work to access health-promoting resources that are increasingly restricted from immigrant communities.

## Research questions

At issue is how Latines navigate a context that functions to restrict access to health-promoting resources. We conceptualized this study in response to calls to deepen understanding of the ways in which immigration and immigrant policies unfold in localized contexts to affect the health and well-being of Latiné immigrant communities, and to examine how communities respond to and negotiate exclusionary immigration and immigrant policies [14, 21]. Additionally, Tuck [63] and other scholars call for a shift away from “damage-centered research” that can have a consequence of reinforcing narratives that characterize communities bearing disproportional effects of oppression as “broken” and as a consequence, can define communities’ relationships with systems of oppression in uni-dimensional ways. Tuck [63] calls for desire-based research that examines responses to racialization processes in ways that avert victim blaming and center the complexities, self-determination, and power of communities. To address this gap, we used qualitative data to examine Mexican-origin women’s responses to a protracted period of policies that constrained immigrant communities’ access to health-promoting resources: Our analysis focuses on women’s individual responses as well as their descriptions of actions taken by and undertaken in support of members of their social networks. Our particular focus was on Mexican-origin women and their co-ethnics (members of their social networks who share an ethnic identity). While our qualitative study initially focused on women of Mexican and/or Central American origins, through our snowball sampling approach (described in the methods section), the majority of participants identified as Mexican-origin, reflecting the largest representation of Latiné-identifying women in Detroit at the time of this study [60]. Accordingly, the findings from this study are specific to the experiences of Mexican-origin first-, 1.5-, and second-generation women at the time of this study. We are interested in women’s agency as they navigated structural factors that restricted their access to a range of health-promoting resources, including their efforts to garner resources to support their own health and well-being and that of other members within their social networks. Our study was conducted during a time (2013–2014) when access to health-related resources was increasingly linked with assessments of legal status. We used a grounded theory analysis of interviews with Mexican-origin women who were immigrants or children of immigrants and who lived in Southwest Detroit, Michigan. By focusing on these dynamics within a northern border community, in this study we sought to deepen understanding of the interplay between efforts to access health-promoting resources and restrictive federal, state, and local policies and practices.

Findings presented in this article are drawn from a study conducted a decade ago that highlighted the intersecting ways in which Mexican-origin communities experienced and navigated exclusionary immigration and immigrant policies and implications for health [43, 50]. The findings outlined in the sections that follow remain relevant given the enduring and magnified context of exclusionary policies and rhetoric towards immigrant communities, as illustrated by an escalation of deportations under the Trump administration [64], recent restrictions on migrants seeking asylum at the US-Mexico border [65], use of Title 42 to restrict migration in the name of pandemic mitigation [66], and anti-immigrant discourse in the 2024 Presidential election cycle [67]. Furthermore, our motivation to examine individual and collective actions to access health-promoting resources responds calls for research that highlights community agency in the midst of racialization processes [63].

## Methods

### Data collection

We conducted interviews in 2013–2014 with first- (*n* = 25), 1.5- (*n* = 10), and second-generation (*n* = 13) Mexican-origin women 18 years of age and older who lived in Southwest Detroit, Michigan and were fluent in Spanish or English (Table 1). First-generation includes Mexico-born women who were 12 years old or older upon migration to the US; 1.5-generation includes Mexico-born women who were less than12 years old upon migration; and second-generation includes U.S.-born women with at least one Mexico-born parent [68].

**Table 1:** Sociodemographic characteristics of study participants: first-, 1.5-, and second-generation Mexican-origin women, Detroit, Michigan

	First generation (*n* = 25)		1.5 Generation (*n* = 10)		Second generation (*n* = 13)	
	% (n)	Median (SD)	% (n)	Median (SD)	% (n)	Median (SD)
Age (years)		45.0 (11.3)		32.8 (14.5)		40.7 (19.0)
Interviewed in Spanish	96% (24)		40% (4)		23% (3)	
High school education or higher	48% (12)		80% (8)		69% (9)	
Employed in formal labor force	8% (2)		60% (6)		31% (4)	
Married or living with partner	96% (24)		50% (5)		38% (5)	
Live in household with 1 + child	88% (22)		70% (7)		77% (10)	
Self-rated fair or poor health	44% (11)		20% (2)		46% (6)	

The research team included staff members of Detroit Hispanic Development Corporation (DHDC), a community-based organization (CBO) in Southwest Detroit, and researchers based at the University of Michigan. Through snowball sampling [69] – a recruitment process by which participants invite other members of their social networks to participate in the research study -- we asked participants and two other local CBOs to share information about the study within their networks. We recruited three-quarters of participants through research team members’ networks and one-quarter through snowball sampling. Participants had the option of completing the interview at their home or at one of three Southwest Detroit CBOs. Two authors (AMWL, CG) conducted semi-structured individual interviews. Topics included: immigrant policies; responses to immigrant policies and sentiments towards immigrants; discrimination; health; and policy recommendations. The interview guide has previously been published elsewhere [43]. The interviewers (AMWL, CG) asked participants for permission to audio-record the interview. Participants completed a brief demographic survey after the interview and received a $20 cash incentive and information about immigrant rights and resources. Interviews lasted from one to three hours and were completed in participants’ preferred language (Spanish: *n* = 31; English: *n* = 17). Two co-authors (AMWL, CG) and research assistants transcribed the interviews verbatim. We did not ask about legal status. However, several participants readily disclosed their legal status when sharing their experiences or those of others in their social networks. We de-identified interviews and use pseudonyms for participants. The first-author translated quotes from Spanish interviews.

### Data Analysis

Through a grounded theory approach, the first-author (AMWL) open coded initial interviews to identify inductive codes [70, 71]. The research team discussed emerging findings to inform the codebook, conducted expert checks, and considered the influence of the research process on participants’ stories. We organized segments of text into codes, grouped codes into categories and themes, applied codes to subsequent interviews, and updated the codebook. We used axial coding to identify connections across codes and categories, and examined patterns within and across interviews [70, 71]. Throughout the process, we iteratively refined the inductive themes, subthemes, or categories by conducting expert checks with the research team and examining the context and patterns within each interview and across interviews [43, 50]. For example, the first-author (AMWL) reviewed the emerging themes and categories and examined the conditions in which these themes and categories emerged, such as by immigrant generation, legal status, and conditions of women’s social networks.

## Results

We identified three major categories to capture Mexican-origin women’s responses to racialization processes as they sought to access and share health-promoting resources (Table 2). These represented individual and collective strategies including: (1) maintaining access to health-promoting resources; (2) limiting activities or contact with stigmatized others in order to protect one’s own identity; and (3) mitigating adverse effects by leveraging social support. In the following sections, we present our analysis of women’s responses, then discuss implications of these responses for maintaining access to health-promoting resources in the context of political, social, and economic barriers.

**Table 2:** Individual and collective strategies to access and share health-promoting resources: categories and subcategories

Categories
1. Maintaining access to health-promoting resources
2. Limiting labeling and stigmatization
3. Mitigating adverse Impacts of Immigrant policing by leveraging social support • Receiving and providing emotional social support • Receiving and providing instrumental social support

### Maintaining access to health-promoting resources

Women used multiple strategies to prevent restrictions on access to resources necessary to maintain health. Those restrictions resulted from immigration and immigrant policies and practices that were grounded in, and that simultaneously reinforced, the construction of a devalued identity. Women in the first- and 1.5-generation recounted several experiences of being asked about their legal status in a variety of contexts. Several immigrant women and women with immigrant family members who had an unauthorized legal status recounted their experiences as they sought to renew their driver’s licenses. For example, Susana, a first-generation woman, shared:

We went all over the place, to all the secretaries [Secretary of State] to see [about renewing driver’s license] and no. We went downtown, we went to … Jackson [Michigan], all over and they said that they were still giving [driver’s] licenses, but we didn’t make it. By the time we went they weren’t [issuing driver’s licenses to undocumented residents]. They didn’t give us one.

A driver’s license is a critical resource that facilitates work and other daily activities: Following implementation of Michigan’s restrictive driver’s license policy, access to this critical resource was limited. This policy both made it more difficult for those with undocumented status to get or renew a driver’s license or state ID and intersected with practices that increasingly required a driver’s license or state ID to access health-related resources. Often, women described going *outside* of Southwest Detroit because they anticipated clerks at their local Secretary of State office would use their Southwest Detroit residence as a racializing marker or symbol of (il)legality [43].

Women reported a variety of results of this strategy at a time when changes in driver’s license access was beginning to have a more widespread effect. Some, like Susana, were unable to renew their driver’s license despite repeated efforts. Others did experience success. For example, Angela, a 1.5-generation woman in her 20’s recalled her mother’s experience:

Before, she [her mother] didn’t have one [a driver’s license] and she went … when they [Secretary of State’s office] had, like, stopped giving them [licenses to undocumented residents], she went and she got it in Taylor, because right here [in Southwest Detroit] they’re more, racist. Like, they know you’re Hispanic so they, right away ask you for your social [Social Security number] and stuff. So, when she went over there and they didn’t ask her for it, she got it [driver’s license] you know, we were like, “Oh, you know, I’m gonna go try it, whatever.” But then after that, I went to go try and I guess, like, they let ‘em know now that you have to have a social [Social Security number], so they didn’t give it to me.

Though she did not have a driver’s license prior to 2008, Angela’s mother’s effort to obtain a driver’s license *after* the implementation of Michigan’s restrictive driver’s license policy highlights the importance of this resource. While not all first-generation women made use of this strategy, several did so, recognizing the importance of a driver’s license in a context in which immigrant policing was pervasive. These efforts also were an attempt to retain access to critical personal and community resources, such as employment, mobility, remaining in the U.S., family cohesion, identifying documents, services, and goods. Without a driver’s license, women experienced heightened social and economic vulnerabilities that have been linked with health inequities [40, 72].

In addition to facing questions about their legal status, second-generation women also recounted experiences in which they felt denigrated based on the racialized identities. For example, Alice, a second-generation woman in her 50’s, recalled her daughter’s experience with a Latina clerk at the Secretary of State’s Office:

Like, um, the Secretary of State that was … here [in Southwest Detroit] that was real bad too, they [clerks] just rolled their eyes just like, Uhh. You know just they did – like they didn’t want to associate themselves with, you know, I’m Mexican or whatever. Like one instance my daughter, they went up to her and they said, “Are you Mexican?”

Despite this experience of having a racialized identity made salient, Alice’s daughter was able to renew her license. Like many other second-generation women in this study, despite discriminatory treatment, she was able to mitigate the effects of racialization through her legal ability to access resources denied to unauthorized immigrants.

Repeated experiences of discrimination, such as those described by women in this study, have been linked to adverse health outcomes among Latinas [73], as well as other racially minoritized groups [74]. Active coping responses, such as those described here related to efforts to obtain driver’s licenses, have been hypothesized to exacerbate those adverse health impacts when they are conducted in particularly restrictive contexts [75, 76] and there is some evidence to support this hypothesis [77].

### Limiting labeling and stigmatization

Women described vigilant efforts to limit labeling and stigmatization by circumscribing their activities in order to prevent surveillance related to immigrant policing. This theme includes strategies to minimize visibility, activities, and mobility in public spaces. For many women, this involved particular care related to driving. As Consuelo, a first-generation woman in her 30’s with an unauthorized legal status, explained:

I always drive with care. To avoid them [police] stopping me. For example, usually every time they [police] call immigration it’s because you um, don’t speak English and you are driving, or you’re speeding or maybe in an accident or you didn’t stop at a stop. So, I try to always drive at the speed limit and always follow the laws that are there so you don’t have any contact with the police.

Consuelo’s accounting illustrated vigilance in a context in which police surveillance was prevalent, reflecting her concern that racial profiling from police and/or minor traffic violations could lead to contact with immigration officials. Consuelo identified her limited English use and, in further conversation, her expired driver’s license, as racializing markers that were leveraged as symbols of (il)legality that heightened her vulnerability to immigrant policing.

Consuelo’s concerns illustrate that vulnerability to immigrant policing derives from immigrant policies that target those who have been racially minoritized. For example, Clara, a second-generation woman in her 40’s whose husband recently became a permanent resident, shares her concern about her husband’s driving experiences:

Even though my husband’s okay, but you still fear. Like he’s gonna be picked up or spotted real easy, like is a cop gonna stop him just for racial profile and just take away his papers? […] That’s why I tell my husband, “Be careful, you know, who you’re with. Just make sure you don’t bring people in the truck, because you don’t know … Because you are a target.” … And um, a couple of times when he’s driving, we’ll have cops behind us. It’s my truck. Has my plates. It says my name on it too. And, it’s like, wait a minute. But, when I drive, nobody bothers me. It’s – it’s because of who’s driving.

Clara’s vigilance centered on her husband’s vulnerability to immigrant policing when driving, whether or not she accompanied him. She was attentive to the possibility that police would “target” her husband, resulting in an encounter with immigration officials, regardless of whether the traffic stop occurred as a result of a traffic violation. Clara’s high level of mistrust in officials was apparent in her narrative, despite her husband’s authorized legal status.

The extent to which women were vigilant depended upon their own vulnerabilities and those of others within their social networks. For women in the first- and 1.5-generations, having an unauthorized legal status or a close relationship with someone with a more vulnerable legal status heightened their vigilance. Among second-generation women, having family members who had an unauthorized legal status contributed to greater intensity and chronicity of vigilance. Driving was the most common – but not the only – context in which women described being vigilant. Women described engaging other vigilant strategies, such as limiting their activities outside of their home such as in public spaces or ceasing to work. For example, Lily, a first-generation woman in her 40’s who has lived in the US for 28 years described restricting when she would leave her house to prevent interactions with immigration enforcement officials: *“You have to think about what can happen outside … The house is surrounded by immigration. This house they are watching me, watching me […] but if I go out when they are outside they are going to grab me.”*

Women described restricting their driving by limiting the frequency, distance, or boundaries in which they drove, and in some cases, ceasing to drive altogether, in response to the heightened surveillance and stigmatization they experienced around driving. For example, Ava, a 1.5-generation woman in her 30’s, described how she stopped driving after her license expired and her sister was deported after a traffic stop:

When I saw that they stopped her [sister] and she didn’t have a license and they took her to immigration - well I said, then I won’t drive […] what would happen with the children? Where would we leave them? And my parents aren’t here, they are in California and they, well no, they have more children and I don’t think they could take care of more children. So yes, you could say I am here alone and I can’t be taking advantage and driving like it’s nothing if you don’t have a license too.

Ava chose to stop driving to mitigate the threat of immigrant policing. Weighing her more limited mobility against the lack of options to care for her children if she were detained or deported, she concluded that a decision not to drive was central to protecting and fulfilling her role as a caregiver.

The decisions women made with regard to driving were contingent and negotiated within particular social relationships. Like Ava, above, Dalilia, a 1.5-generation woman in her 20’s also described her decisions about driving as linked to her role as a caregiver. However, her particular circumstances led to a different decision, albeit one that she described in conflicted terms:

They [immigration officials] told me not to drive and I lasted a while … [I was] really scared and I didn’t drive. I lasted like … one or two months and I couldn’t do it anymore because it’s really difficult not driving. You know here how it is for a mother you have to take your kids to the doctor, you have to – I was working and, and you need transportation you can’t, you can’t be asking people for help all the time because people aren’t going to help you. So I had to – that order I did have to disobey and I had to drive. […] And now every time I see a police in the street I get scared. This didn’t use to happen; now I do get nervous and… it’s difficult….

Dalilia’s decision to stop driving and then to begin driving again reflects the dynamic nature of these decisions, and the ways in which individual decisions are contingent upon the social resources that she could call upon and her caregiving and employment responsibilities. She linked her ability to stop driving to the burdens imposed on network members for rides. Her description highlights the social relationship costs involved in such decisions and balancing the potential strain on those relationships. As she noted, her decision to resume driving simultaneously eased the strain on her social relationships, while increasing her risk of encountering immigration officials. She clearly articulates the costs, in terms of heightened vigilance and stress. The time and energy that women dedicated to frequenting multiple Secretary of State’s offices in order to obtain or renew a driver’s license, as described earlier, highlights the critical role of the driver’s license in accessing health-related resources, such as caregiving and employment as illustrated in women’s experiences noted above.

The health impacts of the strategies women used in response to heightened policing are complex, and deeply intertwined with their social, economic and legal circumstances. Women with more limited social and economic resources and greater vulnerability to immigrant policing (due to either their own status or that of family members) may be more likely to circumscribe their activities in a context of heightened surveillance. While this strategy may reduce the likelihood of encounters with immigration officials, it was clearly balanced against its impacts on broader social networks. Given substantial literature documenting that access to and receipt of social support is health protective [58, 78], women’s concerns about overburdening or fraying those social ties by too great a dependency on them are reasonable. The excerpts above clearly illustrate the negotiated and contingent nature of the decisions women made as they sought to retain access to critical social and material resources (e.g. driving children to school, accessing health care). While constraining, women recognized that their need to rely on others to drive them and their children can simultaneously undermine the social relationships necessary to protect health. In the following section, we examine in greater depth the importance of those social relationships as resources that can be leveraged within racializing contexts.

### Mitigating adverse effects of immigrant policing by leveraging social support

Women described using or providing social support to reduce the effects of racialization and provide resources for identity support. Women who had an unauthorized legal status, lacked a valid driver’s license, experienced family separation through deportation, managed caregiving and/or employment responsibilities, and/or experienced economic hardship were more likely to describe using this strategy. This theme emerged in two categories: emotional and instrumental social support.

#### Emotional social support

Actions in the category of emotional social support included talking with or listening to family or friends about stressors that women were experiencing. Women who had an unauthorized legal status or who had family members who had an unauthorized legal status used this strategy to alleviate the consequences of racialization. Some women discussed these stressors with trusted others. For example, Alicia, a 1.5-generation woman whose husband had an unauthorized legal status, shared, *“I have about two people in my life that I like to vent with and they can advise me very, very wisely. […] I think it takes a load off your shoulders because you’re not stuck with that in you.”* For Alicia, talking with trusted others (e.g., friends, family members) provided an opportunity to discuss stressors related to her husband’s legal status, and her concerns about family separation if he were to be deported. Women also discussed efforts to protect their family and manage their identities. Alicia’s metaphor that talking with others took *“a load off of [her] shoulders”* suggests the physical relief of sharing her experiences and concerns with trusted others. Receiving emotional support may be health enhancing as she and her family contend with racialization processes and their implications. Women’s experiences of drawing support from co-ethnics illuminate the nuances of responses to racialization processes. Specifically, these experiences highlight ways in which co-ethnics may offer unique elements of emotional support, particularly when discussing identities and racializing markers that have been highly stigmatized and/or topics that are highly sensitive and may need to be held in confidence, for which other co-ethnics may have keen understanding through their own lived experiences. Though several women limited their contact with peers to prevent othering and immigrant policing, others discussed their experiences with racialization with trusted members of their social networks, and – like Alicia – described experiencing affirmation and a sense of reprieve in doing so. That relief may be health enhancing.

Accounts of drawing on or providing emotional support were less common than descriptions of providing or receiving instrumental support (described below). The limited discussion of emotional support may reflect several factors. Emotional support may be less visible (and therefore discussed less), although embedded in instrumental forms of support – such that the support women described provided not only critical instrumental support, but also emotional support (e.g., discussing difficult topics during car rides). It is also plausible that other strategies women used to mitigate the adverse effects of racialization, such as limiting mobility and contact with peers, may reduce contact with those to whom women could provide or from whom they might receive emotional support.

#### Instrumental social support

The category of instrumental social support included examples in which women described giving or receiving tangible forms of assistance or support, generally with family or network members. Examples included: giving rides to persons who may lack a driver’s license, receiving rides from someone with a driver’s license, registering cars in the name of a person with a more protected legal status, translating or assisting others with navigating confusing immigrant policies and systems, and providing or receiving housing. Assistance with driving-related concerns (e.g., offering a ride, registering cars) was the most common form of instrumental support received or offered. For example, Bella, a 1.5-generation woman in her 20s, explained that before she received Deferred Action for Childhood Arrivals (DACA) status, she relied on her mother and others to drive her to fulfill her employment responsibilities:

It was very hard. Well … my mom didn’t work at that time so she’d be able to take me here and somebody would have to pick me up or I’d get a ride home from somebody, so I was more home than I was out because I didn’t have a driver’s license. And now it’s different. Now, it’s like you don’t see me at home, I’m always working and so I’m out. …. Before that [getting a driver’s license through DACA] I never drove or nothing because I was like paranoid, and it goes from the cops getting you to asking for your license and it just goes bad from there … I didn’t want to risk it so I was just like, I’ll just stick to getting rides you know – ‘cause I worked around the area.

These forms of assistance helped reduce Bella’s concerns about encountering police or immigration officials while driving to or from work. The family members and friend who provided those rides illustrated their willingness to extend the benefits of their more protected statuses (e.g., driver’s license) to members of their networks who were more vulnerable to adverse consequences of interactions with police or immigration officials. Importantly, those benefits included offering rides for women as they sought to fulfill caregiving and employment responsibilities in a region with a historically underinvested public transit system and limited walkability [79].

Support in the form of rides also enabled engagement in social settings for women who limited their mobility because they lacked a driver’s license. For example, as Susana, a first-generation woman, explained:

When I am [riding] with someone that is good, that is legal, that has their papers, it’s like then I do feel safe, as I say, “Well, I am with her, they won’t do anything to me, they won’t say anything to me.” [laughs] And it’s the same though either way they [police or immigration officials] won’t say anything to us. [laughs] No they won’t do anything! But yes, I do feel safe.

This provision of a ride enhanced Susana’s mobility, facilitated interactions with friends, and avoided the isolation that otherwise would have come with the decision to not drive without a license. Thus, instrumental support enabled social participation and offered one gateway to the social relationships essential to both emotional and instrumental support. Several women who had driver’s licenses recounted providing rides to family members, friends, or neighbors, illustrating the mutual social relationships that enabled women to support each other as they sought to respond to discrimination associated with racialized immigration status.

Women with protected statuses (e.g., having a driver’s license, DACA status, or another authorized status) also described sometimes registering cars for others in their own name, and ensuring that insurance payments were up-to-date on vehicles used by those without a license. Alicia, a woman in the 1.5-generation, explains the strategic actions she took to protect her husband during the time that he lacked authorized legal status:

I always make sure the taillights work on my car. I always make sure that things are so perfect. I have insurance … And there are times I wanna give up on that $400 payment for all three vehicles. But the fact that I know that that *might* [emphasis] reduce the risk of *him* [emphasis] getting in trouble is worth those $400 a month.

Indeed, several women provided similar support for their family, registering or insuring vehicles for them, despite the economic hardship that entailed.

Some first- and second-generation women extended their home to family or friends who experienced a deportation and/or a financial hardship related to legal status, immigrant policing, and exclusionary immigrant policies. For example, Margarita, a woman in the first-generation took in a friend’s daughters after her friend was deported. She explained: *“Because from the moment [they asked if they could stay with her] I said: where one eats, two can eat. Where three eat, four can eat.”* Despite their own economic struggles, women sought to protect and support their network members by adopting economic and family responsibilities once borne by those who were deported and/or who struggled to make ends meet.

Women in the first- and 1.5-generations also expressed concerns that asking for emotional or instrumental support would burden their networks. Ava, a 1.5-generation woman, describes trying not to bother anyone:

Since [my license] expired I hardly ever drove because it was hanging over me and sometimes I needed to drive, like, the child to the doctor or things like that where I needed to leave to buy things … I felt bad because well I don’t like bothering anyone and well I had to keep bothering people. […] many times I didn’t want to ask anyone because I felt bad bothering people or sometimes they tell you no and I feel bad when they say no to me. So sometimes to avoid them telling me “no” I wouldn’t ask anyone ….

Several other women echoed Ava’s fears of possible rejection when requesting support from others. Of note, women who had an unauthorized legal status, family members with a less protected legal status, and/or limited networks (e.g., single parents, no extended family in the area) were most likely to express this concern. This hesitation may be because they are least able to reciprocate or may need to make more requests for assistance and thus are particularly sensitive to not overburdening their networks.

These worries suggest that the forms of social support available may depend on the number and strength (e.g., time invested, intensity, and intimacy) of the relationships or social ties between those giving and receiving support [80]. Granovetter [80] theorized that weak social ties are important for expanding social networks and accessing informational support that may not be embedded within the networks of stronger social ties. In the present study, although some 1.5- and second-generation women who had a valid driver’s license registered other people’s cars in their name and insured those cars, none did so for non-family members. Ava’s inability to find someone to register her car may reflect the structure of her local social network. For example, earlier in the interview Ava explained that her decision to stop driving after her driver’s license expired was motivated by her concern that she had no local family to care for her children if she were detained or deported. Thus, because this form of support is potentially expensive and risks the registrant’s personal insurance record, women with local family members may be better able to draw on such support. Separately, registration of a car for a family member enabled that family member to be more mobile, independent, and to fulfill caregiving and employment responsibilities, some of which may be mutually beneficial within the family. Relatedly, women described giving rides to, or receiving them from, members of their social networks with whom they had strong ties or weaker though trusted relationships (e.g., neighbors). Based on this example, it is plausible that women may find it easier to ask for, or provide, instrumental support that entails substantial financial commitment with members of their family, or others who are highly trusted. Women who needed to draw upon such instrumental support frequently, particularly if they are not in a position to reciprocate, may experience strains in their social ties. Given the chronicity with which women needed support with driving, women may need to tap multiple weak ties so as not to stretch any one relationship too thin.

## Discussion

This study sought to understand how Mexican-origin women in a midwestern northern border community navigated and responded to racialization processes within the context of exclusionary immigration and immigrant policies to reduce adverse effects and care for themselves, their family, and their community’s well-being. Women’s active efforts to navigate these dynamics, and those they described being taken by family and friends, were dynamic and shaped both by their own immigration experiences and the resources on which they could draw in shaping their responses. Women acted intentionally and strategically to buffer the consequences of policies and processes that limit access to health-promoting resources, circumscribe mobility and contact with others, and stigmatize their identities. Their actions were undertaken within dynamic relationships, in which some actions to reduce adverse effects of racialization (e.g., limiting activities) had subsequent implications for the social networks that were so central to their ability to engage social resources to help prevent or mitigate the social, economic, and health vulnerabilities they experienced.

This study builds upon the literature on the impact of immigration and immigrant policies on Latiné communities by highlighting how women actively negotiated these processes, including: attempting to access resources that are restricted as a result of racialization, limiting their activities to prevent exposure to immigration enforcement apparatuses, and leveraging social support to offset the health consequences of racial exclusion. The findings presented here suggest that women’s experiences of, and responses to, the policies that constrict access to resources varied according to immigration-related factors (e.g., legal status of self or kin network members, family separation) and resources upon which they could draw.

The proactive efforts of women and those in their networks to secure a current driver’s license highlight how policies that restrict access to state IDs and driver’s licenses have become immigrant policies that regulate access to health-relevant resources. Other scholarship has demonstrated the importance of having a current driver’s license to accessing occupational opportunities, identifying materials (e.g., birth certificates), economic resources, health care, safety net programs, and goods and services linked with health [19, 32, 40]. Women’s persistence in attempting to get or renew a driver’s license illuminates their active, effortful attempts to secure health-related resources, which could have implications for health over the short-, intermediate-, and longer-term. For example, successful efforts to secure a driver’s license may facilitate mobility and access to economic, occupational, social, and material resources for the duration of the license, resources which can in turn be shared with social network members. Unsuccessful attempts to renew or get a driver’s license may further limit both mobility and access to health-promoting resources, which are increasingly contingent upon having a current government-issued ID. Additionally, attempts to get or renew a driver’s license that are met with racist inquiries or comments may exacerbate race-related stressors and health inequities.

These negative experiences and acts of questioning eligibility for a driver’s license illuminate the ways in which government-issued ID policies and bureaucratic processes constitute a racially biased system and how interactions with a governmental institution or negative actors within that institution can negatively affect access to health-promoting resources [41]. Moreover, women’s responses to these race-related stressors reflect the dynamic nature of racialization processes, as illustrated by women’s significant efforts to assert and affirm their power and agency in the face of stigmatizing and dehumanizing policies, discourse, and symbolic and interpersonal interactions. The minority stress model posits that racially minoritized persons and communities may experience stressors captured by stress process frameworks, as well as stressors that are unique to persons who are minoritized through racist structures and interactions, which may heighten vulnerability for adverse health outcomes [81, 82]. The minority stress model also recognizes that communities disproportionately affected by racialization processes may leverage individual and collective strategies to manage or address these stressors [81, 82]. Findings from the present study highlight several key mechanisms by which Mexican-origin women navigate race-related stressors in the midst of restrictive twenty-first century immigration and immigrant policies, and how they leverage social and tangible resources and strategies to interrupt the effects of structural racism.

### Social support and social networks

In the present study, women’s accounts suggest that the mechanisms by which social support may affect health are complex and shaped by their social networks and other resources (e.g., driver’s license). Further, women’s and their network members’ ability to draw on social support to mitigate the effects of racialization were contingent upon the other resources that they engage, which are also shaped by their vulnerabilities and protections (e.g., legal status, social networks). Subsequently, the effect of social support on health may be influenced by their vulnerabilities, protections, and other responses and resources that they and their network members leverage.

A sizable literature indicates that social support is protective of health [58, 83], including salubrious health outcomes among Latines [84]. Findings from this study suggest that social support is multi-dimensional and that the mechanisms by which social support intersects with racialization responses to affect health may vary according to the forms, sources, and availability of social support. These findings support previous research linking closeness to social network members and/or a sense of isolation to perceiving, accessing, or providing support [85, 86].

One way that social support is associated with health is through stress buffering effects [87, 88]. Findings from the present study align with a literature indicating that social support may reduce the adverse health implications of racialization [89, 90]. Instrumental social support may promote health by enabling women and their network members to earn a living and fulfill other responsibilities, a finding that is consistent with literature conceptualizing instrumental support as critical source of assistance with concrete issues in day-to-day life [91, 92].

Additionally, instrumental support may enhance health by providing opportunities to engage with others [93], particularly when other responses to racialization processes may limit social connections. For example, findings from this qualitative inquiry suggest that instrumental support may buffer the potential health consequences of other responses to racialization, such as circumscribing activities and mobility and/or restricting contact with peers. Thus, women’s active efforts to navigate the demands that they placed on their networks for instrumental or emotional support may, in and of themselves, be health protective as they work to preserve the networks themselves. Those with more limited social networks may have reduced ability to access social support [86, 94], thus limiting the very resources available through those networks to mitigate adverse effects of racialized immigration and immigrant policies.

A substantial body of evidence suggests that *receiving* social support can be health enhancing [58, 78, 95]. A limited body of cross-sectional and longitudinal studies suggest that giving social support is correlated with health enhancing outcomes [96–98]. However, increased risk of adverse health outcomes in older age [99, 100] may limit the ability to provide social support, suggesting the possibility of a more complex, inverse association of health and social support across the life course. Women in the present study described being able to support others as health-promoting. For example, providing emotional or instrumental support may be a response to inequities stemming from systems of exclusion in an effort to care for one’s self and community members [92, 101, 102]. Other evidence has indicated that both the provision and receipt of social support is contingent upon the context and resources on which women can draw [54]. For example, in the present study, some women were concerned about bothering others and therefore were highly selective when they asked for support. Other women who took in family members affected by immigration policies also adopted caregiving and financial responsibilities that could be sources of economic vulnerability and stress, which has important implications for health given the literature linking financial stress with adverse health outcomes [103, 104].

This qualitative inquiry identified several mechanisms through which social networks may affect health in the context of racialization processes, as well as feedback loops and potential dampening or exacerbating effects. The social support literature generally conceptualizes social relationships and support networks along three dimensions: social network structure, interactional characteristics between the person and network, and function of network members. Members may provide or utilize support such as emotional, appraisal, informational, and/or instrumental support; network growth; and affirming and/or preserving social identities [105, 106]. In the present study, social networks influenced the range of options available to women as they sought to prevent, buffer, and respond to racialization processes. Women with smaller social networks described a more limited range of responses that they could utilize for emotional and instrumental support. Overreliance on those thin networks risked weakening these sources of support, thus creating a feedback loop that risked reducing their networks even further. The nature and strength of women’s social relationships shaped their responses to racialization processes, such as limiting activities and leveraging social support. Stronger social relations, such as those between some family members and friends, may be critical for emotional support and certain forms of instrumental support. In contrast, Granovetter [80], in his concept the “strength of weak ties”, posited that weak social connections are critical for the growth of social networks and informational and instrumental support. Our findings suggest that under contexts of significant systematic exclusion – such as exclusionary processes linked with racialized immigration and immigrant policies – women in this sample described infrequently relying on weaker ties, while they generally described greater reliance on or provision of social support to family and kin networks (stronger ties).

Although social support influenced some responses to racialization, racialization processes may have also influenced social networks and relationships. For example, restricting interactions with peers may affect the structure of women’s networks (e.g., strong and weak ties) and the social resources available to engage emotional or instrumental support. Some women recalled giving or receiving rides to or from weaker ties such as neighbors or acquaintances. Limiting social interactions to mitigate the effects of racialization may constrain the ability to engage *other* responses (e.g., support to conduct employment and caregiving responsibilities). Thus, several responses to racialization intersect with the social relationships available to women and in which they have invested or are able to invest. These approaches highlight the importance of understanding social networks and health as dynamic systems comprised of reciprocal, multifaceted relationships and responses.

### Limitations, strengths, and future research

This study has some limitations. Findings are based upon in-depth interviews with Mexican-origin women in a low-to-moderate income northern border community during a period (2013–2014) of increasingly exclusionary immigration and immigrant policy context in the U.S. Findings from this study should be interpreted in a manner that contextualizes them within this community, immigration context, and period of inquiry. Additionally, the findings reported here should be understood in the context of the interview’s discussion topics and the time period (2013–2014). Given our study’s focus on women’s experiences with immigrant policing, women might experience other racialization processes that were not discussed during the semi-structured interview. Further, research is warranted that tests the reported mechanisms by which social networks and social support may shape efforts to prevent, buffer, and respond to racialization processes and implications for health. Given that anti-immigrant and anti-Latiné sentiments and policies increased substantially in the years following this study (e.g., Presidential election discourse, 2016–2020 Presidential term, COVID-19 pandemic response, restrictions on migration of asylum-seekers in 2024), future research is warranted pertaining to recent and/or ongoing aspects of exclusionary immigration (e.g., detention, restricting asylum seeking processes) and immigrant policies (e.g., public charge) [22, 65]– [67, 107]. Additional research is needed on the health impacts of the need to exert high effort to obtain the resources in the face of institutional barriers such as exclusionary immigration and immigrant policies. Moreover, studies are needed that examine these dynamics in greater depth for men, transnational social ties, and comparative analyses of other Latiné subgroups (e.g., Central American, South American, Caribbean subgroups) who are affected by similar policies to provide insight into the unique and shared strategies to access health-promoting resources.

This study also has several strengths, including the case study approach to examining responses to racialization and health implications, an examination of how community dynamics shape efforts to access and share health resources, and the intersectional analysis of the role of multiple social statuses (e.g., legal status, caregiving status) in shaping responses to racialization.

Despite the fact that the data for this analysis were collected a decade ago, findings reported here remain salient. Anti-immigrant rhetoric and policies of the U.S. have escalated over the past decade, with growing calls for expanded immigration enforcement activities during the 2024 Presidential election cycle, and the nationwide implementation of the REAL ID ACT (previously experienced as a patchwork of state-level responses to this federal policy, and for which implementation deadlines have been delayed several times) that will take effect across the U.S. by 2025. There is growing urgency to understand both the underlying drivers of these anti-immigrant efforts and to detail their health impacts. Given the protracted and growing nature of exclusionary immigration and immigrant policies in the U.S., future studies that examine the implications of such policies and social environments for the well-being of immigrant communities are increasingly important.

### Implications

The findings presented here underscore the ways that racialized policies and practices constrain access to health-promoting resources. The dynamic processes that racialize immigrant women and circumscribe their access to health-promoting resources are particularly important in the current political moment, as anti-immigrant sentiment and policies escalate, and as Latiné immigrants increasingly disengage from civic life and services [108]. These findings also illuminate the role of legal status and social networks in shaping women’s efforts to maintain access to those resources, as well as their efforts to mitigate the adverse impacts of their loss. Together, these findings suggest two opportunities to address the racialization processes that underlie restrictive immigration and immigrant policies, and to mitigate their adverse effects on health.

First, Mexican-origin women with an unauthorized legal status experienced restricted access to health-promoting resources. The impacts of exclusionary immigration and immigrant policies extend beyond individuals whose legal status is questioned to impact family members. They point to the health-promoting potential of a shift away from immigration enforcement and exclusionary immigrant policies toward strategies that provide viable and timely pathways to citizenship and promote the full incorporation of immigrants (e.g., enabling driver’s license access regardless of legal status) [42, 109]. For example, state-level policies that enable immigrants to obtain or renew a government-issued ID, such as commonly recognized driver’s licenses and state IDs, would enable immigrants and their social networks to access a host of health-promoting resources that are increasingly contingent upon having a US government-issued ID [32, 41]. Likewise, institutions (e.g., community health centers, food banks, social service providers, financial institutions, schools) could improve access to health-relevant resources by reviewing policies and protocols to identify any exclusionary practices (e.g., requiring a current driver’s license from a US state) and implementing alternative inclusionary policies (e.g., accepting any identifying documents that affirm the identity of the resident or client) [32, 41]. Findings reported here suggest that creating more inclusionary immigrant policies at state and institutional levels hold promise for improving outcomes at individual, family, and community levels.

Second, these findings highlight the critical importance of social networks for offsetting restricted access to health-promoting resources and mitigating policies or practices that limit individual mobility. Social networks offer emotional, economic, and other tangible resources for both immigrant and U.S.-born co-ethnics and are particularly important for stigmatization and restricted access to health-relevant resources linked to immigration and immigrant policies. Findings from this study crystalize the ways that immigrant policies can undermine or erode social networks as, for example, some individuals circumscribe social activities to avoid exposure to immigrant policing. Similarly, public charge rules that potentially penalize immigrants who access public assistance while they seek citizenship can create additional economic strains that may in turn further fray social networks [110, 111].

Addressing the punitive public policies that underlie these processes can protect the health of Mexican-origin women and their families both directly by maintaining access to critical resources, and indirectly by averting tensions and strains that both weaken health-promoting social networks and are associated with poorer physical and mental health status. Community-based initiatives may be harnessed to support this segment of immigrant communities in an effort to expand access to social networks, which may improve access to and the exchange of emotional, tangible, and informational forms of support [80, 105, 106], which in turn are associated with enhanced health status.

## Data Availability

The datasets generated and/or analyzed during the current study are not publicly available to protect the privacy of participants and due to safety concerns regarding immigration enforcement. De-identified, available themed data can be made available from the corresponding author on reasonable request.

## Declarations

### Authors information

Alana M.W. LeBrón is Associate Professor of Health, Society, and Behavior and Chicano/Latino Studies at the University of California, Irvine. Her scholarship focuses on mechanisms by which structural racism shapes the health of communities of color, with a focus on policies, systems, and environments. Much of her scholarship involves community-based participatory research, working in partnership with members of affected communities to strengthen understanding of the ways in which structural racism shapes health inequities and to develop and evaluate strategies to advocate for structural change, mitigate the health impacts of structural racism, and create new systems to promote health equity.

Amy J. Schulz is University Diversity and Social Transformation Professor of Health Behavior and Health Education at the University of Michigan School of Public Health. Her research focuses on social and physical environmental factors and their effects on health, health equity, and urban health. Much of her research is conducted with partners in Detroit, Michigan, using a community-based participatory research approach to understand and address drivers of excess cardiovascular risk through the Healthy Environments Partnership, conduct health impact assessments of proposed policies, and develop public health action plans to reduce air pollution and promote health in Detroit and surrounding areas.

Cindy Gamboa is Community Organizing and Advocacy Director at the Detroit Hispanic Development Corporation. A longstanding resident of Detroit, she has been serving the Southwest Detroit community for over a decade and received her bachelor’s degree in sociology and psychology from the University of Michigan at Dearborn. Previously, she was the Community Coordinator for the Healthy Environments Partnership, a longstanding community-based participatory research partnership working to understand and address excess cardiovascular risk in Detroit and was active in several collaborations, programs, and policy change initiatives to promote health equity.

Angela Reyes is Executive Director and Founder of the Detroit Hispanic Development Corporation. Born in Southwest Detroit, Michigan, she brings expertise in critical policy issues that affect residents of Southwest Detroit and other urban areas, including positive youth development, youth violence, substance abuse, immigration, educational reform, community organizing, and community-based participatory research. She received her Master’s in Public Health from the University of Michigan and is a founding and active member of the Healthy Environments Partnership.

Edna Viruell-Fuentes was Associate Professor of Latina/o Studies at University of Illinois Urbana-Champaign. Her research focused on Latina/o/é and immigrant health; transnational processes, practices, and methods; and structural determinants of health contexts, including neighborhoods and immigration policies.

Barbara A. Israel is Professor of Health Behavior and Health Education at the University of Michigan School of Public Health. She has published widely in the areas of social and physical environmental determinants of health and health inequities; the relationship among stress, social support, and physical and mental health; and community-based participatory research. Since 1995, she has worked with academic and community partners to establish and maintain the Detroit Community-Academic Urban Research Center (Detroit URC), which works to foster and support the development of equitable community-academic partnerships focused on understanding and addressing health inequities in Detroit.

## Abbreviations

CBO: Community-Based Organization
DACA: Deferred Action for Childhood Arrivals
